# CircRNA amyloid precursor protein by competitive adsorption of microRNA-6838-5p mediates CDV3 expression to enhance malignant behavior and Warburg effect in Gastrointestinal Stromal Tumor

**DOI:** 10.1016/j.clinsp.2024.100423

**Published:** 2024-07-02

**Authors:** Zhaorigetu, ChunJuan Wang, XianJing Zeng, JinHua Yuan

**Affiliations:** a Department of Gastroenterology, The Affiliated Hospital of Inner Mongolia University for Nationalities, Tongliao City, Inner Mongolia Autonomous Region, 028000, China; b Health Management Center, Yantai Qishan Hospital, Yantai City, Shandong Province, China; c Department of General Practice Medicine, Affiliated Hospital of Jinggangshan University, Ji'an City, Jiangxi Province, China

**Keywords:** CircAPP, miR-6838-5p, CDV3, Gastrointestinal stromal tumor, Metastasis, Warburg

## Abstract

Dysregulated circular RNA (circRNA) expression profiles and their carcinogenic contributions have been noted in nearly all human cancers. This study aimed to unearth the role of circRNA Amyloid Precursor Protein (circAPP), an abnormally highly expressed circRNA in Gastrointestinal Stromal Tumors (GIST). As the results found, circAPP was upregulated in GIST tissues and cells. FISH experiment, dual-luciferase reporter experiment, and RIP experiment confirmed that circAPP promoted CDV3 expression by absorption of miR-6838-5p in GIST. Cell experiments confirmed that silencing circAPP inhibited GIST cell proliferation, migration, invasion, glucose consumption, lactate production, ATP level, expression of HK2 and PKM2, decreased ATP/ADP, and increased NAD^+^/NADH, but promoted apoptosis, whereas overexpression of circAPP did the exact opposite. Furthermore, miR-6838-5p depletion and CDV3 overexpression abolished the influences of downregulating circAPP and overexpressing circAPP on GIST cells, respectively. Animal experiments displayed that circAPP knockdown inhibited GIST tumor growth and liver metastasis. All in all, circAPP promotes GIST cell proliferation and the Warburg effect by miR-6838-5p/CDV3 axis and circAPP may be a potential future therapeutic target for GIST.

## Highlights

• Knockdown of circAPP inhibits GIST proliferation and the Warburg effect.
• miR-6838-5p induces GIST proliferation and the Warburg effect.
• CircAPP activates GIST proliferation and the Warburg effect by regulating the miR-6838-5p/CDV3 axis.

## Introduction

Gastrointestinal Stromal Tumor (GIST) occurs mainly in the stomach and small intestine but also in the colon and rectum, esophagus, and other organs.^1^ Mutations that activate Tyrosine Kinase (KIT) or platelet-derived growth factor receptor α are reported to be the main molecular mechanism of GIST.^2^ Currently, KIT inhibitor (imatinib) is the first-line chemotherapeutic drug for GIST treatment, but most GIST patients develop imatinib resistance.^3^ Although key aspects of the pathogenesis of GIST have been elucidated, the mechanism of oncogene overexpression and gene expression regulators in GIST remains unclear. Metabolic reprogramming is the earliest known cancer hallmark, which achieves tumor cell proliferation and survival by altering bioenergetic and biosynthetic pathways, and tumor cells exhibit a high rate of glycolysis, also known as the Warburg effect.^4^ Therefore, exploring the regulatory mechanism of the Warburg effect is likely to develop new strategies for GIST treatment.

CircRNAs are endogenous non-coding RNAs that differ from traditional linear RNAs in that they have covalently closed structures.^5^ CircRNA/microRNAs (miRNAs) interaction can regulate gene expression and participate in various physiological and pathological processes.^6^ Furthermore, due to the high stability and conservation of circRNAs and their high abundance in body fluids, circRNAs have been considered potential biomarkers for GIST.^7^ CircRNA Amyloid Precursor Protein (circAPP) is an abnormally highly expressed circRNA in GIST.^8^ In the preliminary experiments, it was also determined that circAPP was abnormally highly expressed in GIST. Therefore, this study focused on exploring the function and potential downstream molecular mechanisms of circAPP in GIST.

CircRNAs expressed in the cytoplasm contain miRNA binding sites to act as miRNA sponges, preventing miRNAs from interacting with mRNAs in the 3′Untranslated Region (UTR), thereby modifying miRNA downstream mRNAs, thereby affecting a series of cellular activities.^9^ miRNAs are capable of modifying post-transcriptional gene expression by recognizing and binding to the 3′-UTR of target gene mRNAs.^10^ Dysregulated miRNAs are key to many cancers including GIST, where they act as tumor suppressors or oncogenes.^11^^,^^12^ For example, miR-4510 promotes GIST cell invasion and metastasis^13^ whereas miR-494 suppresses GIST development.^14^ miR-6838-5p has been confirmed to be involved in osteosarcoma,^15^ gastric cancer, and renal cell carcinoma.^16^^,^^17^ However, no studies have demonstrated its actions in GIST.

Targeting to unearth the mechanism of circAPP in GIST, various studies were included, ultimately discovering the mediatory impacts of circAPP on proliferation, migration, and Warburg effect through miR-6838-5p targeting CDV3 in GIST, and hopefully replenishing the options for molecule-targeted therapy in GIST.

## Materials and methods

### *Clinical samples*

Between April 2013 and July 2015, 52 pairs of GIST tissue samples and adjacent normal tissue (≥ 5 cm from cancer tissue) were obtained from the Affiliated Hospital of Inner Mongolia Minzu University. Inclusion criteria: 1) All the included subjects met the diagnostic criteria of GIST according to the criteria of “Chinese Expert Consensus on Diagnosis and Treatment of Gastrointestinal Mesenchymal Tumor (2017 Edition)” and “NIH2008 Modified Chinese Consensus 2017 Revised”; 2) Patients with GIST observed by abdominal CT or ultrasound endoscopy with tumor located in the stomach, and those who were diagnosed by pathology; 3) Clinical data of all the study subjects must be accurate and complete; 4) Patients not undergoing any radiotherapy or chemotherapy; 5) Patients with no history of other concomitant malignant tumors. With written informed consent, tissue sampling was done with the approval of the Ethics Committee of the Affiliated Hospital of Inner Mongolia Minzu University (n° 201211Y62). After diagnosis by a pathologist, tissue specimens were frozen in liquid nitrogen and then stored at -80 °C to determine gene expression. All methods in the study were carried out in accordance with the ARRIVE guidelines.

### *Cell culture*

The human gastric mucosal epithelial cell line GES-1 and the human GIST cell lines GIST-882 and GIST-T1 (Cosmo, Tokyo, Japan) were serially passaged for no more than 6 months. Cells were cultured in DMEM (Gibco; Thermo Fisher Scientific) in a humidified incubator at 37 °C with 5 % CO_2_. 10 % heat-inactivated fetal bovine serum (Gibco; Thermo Fisher Scientific), 100 U/mL penicillin, and 100 mg/mL streptomycin were supplementary to the culture medium. The cell lines were all validated by STR profiles and tested negative for mycoplasma contamination.

### *RNA extraction and measurement*

Under the guidelines of TRIzol (Invitrogen), total RNA was extracted. For reverse transcription, the PrimeScript RT kit (Takara, Shiga, Japan) was employed. Taking cDNA as a template, RNA quantification was done using specific primers (Table 1) and TB Green qPCR Master Mix (Takara) on a 480 II instrument (Roche Diagnostics, Basel, Switzerland). Expression levels of circRNA, mRNA, and miRNA were calculated by the 2^−ΔΔCt^ method and standardized to GAPDH or U6.

**Table 1:** Primers.

	Primers
circAPP	Forward: 5′-TGACTGAAGGGAAGTGTGCC-3′
	Reverse: 5′-CAAACTCACCAATGGCGCTG-3′
miR-6838-5p	Forward: 5′-GCAAGCAGCAGTGGCAAG-3′
	Reverse: 5′-GCAGGGTCCGAGGTATTC-3′
GAPDH	Forward: 5′- CACCCACTCCTCCACCTTTG-3′
	Reverse: 5′-CCACCACCCTGTTGCTGTAG-3′
U6	Forward: 5′-CTCGCTTCGGCAGCACA-3′
	Reverse: 5′-AACGCTTCACGAATTTGCGT-3′

Note: circAPP, circular RNA APP; miR-6838-5p, microRNA-6838-5p; GAPDH, Glyceraldehyde-3-Phosphate Dehydrogenase.

### circRNA stability tests

For RNase R treatment, total RNA (2 μg) was cultured with 3 U/μg RNase R (Geneseed Biotech, Guangzhou, China) at 37°C for 10 min. Then, RNA purification was done with the RNeasy MinElute Cleanup kit (Qiagen, Hilden, Germany). For actinomycin D treatment, RNA was treated with 2 mg/mL actinomycin D (Sigma-Aldrich). Dimethyl sulfoxide was taken as a control. Finally, RNA expression levels were quantitatively analyzed.^18^

### *Subcellular isolation*

Subcellular localization of circAPP in GIST-882 cells was analyzed. Nuclear and cytoplasmic fractions were isolated using the PARIS™ kit (Invitrogen), with U6 and 18S rRNA as positive references, respectively.^19^

### *Gene expression modification in cells*

Small interfering RNA targeting circAPP and CDV3, small interfering RNA negative control (si-circAPP: 5’-ATGGATGTTTGCGAAACTCATCT-3’, si-CDV3, si-NC), overexpression plasmid targeting circAPP and CDV3 and negative control (pcDNA 3.1-circAPP/CDV3, pcDNA 3.1) were supplied by RiboBio (Guangzhou, China), whereas miR-6838-5p mimic (5’-AAGCAGCAGTGGCAAGACTCCT-3’)/inhibitor and mimic/inhibitor NC were by Genepharma (Shanghai, China). Lipofectamine 2000 (Invitrogen) served for the transfection of cells seeded at 2×10^5^/well on 6-well plates. Briefly, when cells were 70-80 % confluent, oligonucleotides or plasmids were diluted in Opti-MEM® Medium. Lipofectamine 2000 was then diluted with Opti-MEM® Medium. Lipofectamine 2000 was mixed with the diluted oligonucleotide or plasmid and added to each well at 100 μL/well. After 48h, the transfection efficiency was determined by RT-qPCR or western blot.

### *Determination of colony formation*

Cell samples from log phase cultures were transferred to 6-well plates at 500 cells/well and cultured for 14 days. Afterward, samples were fixed in 4 % paraformaldehyde and counted after 0.1 % crystal violet staining.

### *Determination of proliferation*

GIST-882 and GES-1 cells were incubated with 30 μM EdU (KeyGen Biotech, Jiangsu, China) for 2h and treated with DAPI (Sigma-Aldrich) to stain the nuclear. Fluorescent images were taken to assess the EdU-positive rate.^18^

### *Apoptosis detection*

Apoptotic cells were assessed with an annexin V-FITC/PI kit (Vazyme, Nanjing, China).^20^ Cells were resuspended and stained with Annexin V-FITC and PI solutions (5 μL each solution) in the dark. Apoptotic cells were detected on a FACSCanto II flow cytometer (BD Biosciences) to calculate the apoptosis rate.

### *Glucose consumption, lactate production, and ATP level measurements*

Glucose consumption, lactate level, and ATP were determined by glucose assay kits (Sigma), lactate colorimetric/fluorometric kits (BioVision, CA, USA), and ATP assay kits (Thermo Fisher Scientific), respectively.^21^

### *ATP/ADP and NAD^+^/NADH detection*

ATP/ADP was measured using the ApoSENSOR ADP/ATP Ratio Assay Kit (K255-200, BioVision, CA, USA) and luminescence was measured by spectroscopy (Molecular Devices, CA USA). Cells (10^4^ cells) were seeded onto luminometer plates, incubated with nucleotide release buffer, and added with 1 µL of ATP monitoring enzyme. Data were recorded at 1 min (Data A) and 10 min (Data B). ADP convertase was then added, and values were read (Data C). ATP/ADP=DataA/(DataC−DataB).

NAD^+^/NADH was tested using the EnzyChrom™ NAD^+^/NADH Ratio Assay Kit (E2ND-100, Bioassay Systems, CA, USA). A total of 10^5^ cells were homogenized and cultured with 100 mL NAD extraction buffer for NAD assay or 100 mL NADH extraction buffer for NADH assay. Next, 20 µL of assay buffer was added to and 100 mL of back-extraction buffer was then supplemented. The mixture was then centrifuged, and the supernatant was transferred to the working reagent. OD_565 nm_ was read at 0 and 15 min.

### *Western blot*

Total protein from cells and tissues was isolated using Lysis Buffer (Thermo Fisher Scientific) and analyzed for concentration using the BCA Assay Kit (Bio-Rad). Proteins (30 μg) were separated by 10 % SDS-PAGE and then transferred to PVFD membranes (Millipore, MA, USA). Membranes were blocked with 5 % skim milk, mixed with primary antibodies GAPDH (60004-1-Ig, Proteintech), CDV3 (LS-C205853, LSBio), HK2 (22029-1-AP, Proteintech), PKM2 (15822-1-AP, Proteintech), followed by the secondary antibody (Sigma-Aldrich). To visualize protein bands, a Western blot substrate (Thermo Fisher Scientific) was added, and Image Lab analysis software (Bio-Rad) was employed for reading protein band intensity.

### *Dual-luciferase reporter assay*

CircAPP and CDV3-3′UTR wild-type sequences (WT-circAPP, WT-CDV3) or mutant sequences (MUT-circAPP, MUT-CDV3) containing the miR-6838-5p binding site were cloned into the vector psiCHECK-2. The above-mentioned reporter gene vector, along with miR-6838-5p mimic or mimic NC was co-transfected into GIST-882 cells (5×10^4^) in the 24-well plates using Lipofectamine 2000 (Invitrogen). Cells were lysed after 48h, and luciferase activity was evaluated using Dual-Luciferase Assay kit (Promega).

### *RNA immunoprecipitation (RIP) experiment*

The RIP assay was performed using the Imprint® RIP Kit (Sigma-Aldrich). GIST-882 cells (1×10^6^) were lysed with RIP buffers and interacted with antibody-coated magnetic beads at 4°C overnight. Anti-IgG was the control for Anti-Ago2. After RNA isolation from the magnetic beads, analysis of RNA expression was carried out by RT-Qpcr.^22^

### *Tumor xenografts*

Twenty-four 4-weeks-old BALB/c nude mice (Vital River Laboratory Animal Co., Ltd., Beijing, China) were animal experiment subjects. The experiments were approved by the Institutional Animal Care and Use Committee of the Affiliated Hospital of Inner Mongolia Minzu University (n° 201306YT2). All animal experiments complied with the ARRIVE guidelines. GIST-882 cells (1×10^6^) stably transfected with si-NC and si-circAPP were subcutaneously inoculated into BALB/c nude mice (*n* = 6/group), and tumor volumes were measured weekly with vernier calipers from the second week after injection. Volume=(length×width2)/2. Five weeks later, all mice were euthanized by cervical dislocation under isoflurane (5 %) treatment. Tumors were resected for immunohistochemistry with antibodies against HK2 (22029-1-AP, Proteintech), PKM2 (15822-1-AP, Proteintech), and Ki-67 (ab15580, Abcam).^23^ To determine the distant metastasis of tumors, GIST-882 cells (1×10^6^) stably transfected with si-NC and si-circAPP were injected into nude mice via tail vein, and the nude mice were euthanized after 8 weeks to assess liver metastasis by HE-staining.^24^

### *Data analysis*

All statistical analyses were performed using GraphPad Prism 9.0 (GraphPad Software, CA, USA). Data represent mean ± Standard Deviation (SD). Two-tailed Student's *t*-test and one-way ANOVA determined statistical differences. Statistical significance represented *p* < 0.05.

## Results

### *CircAPP is upregulated in GIST*

**Fig. 1:**
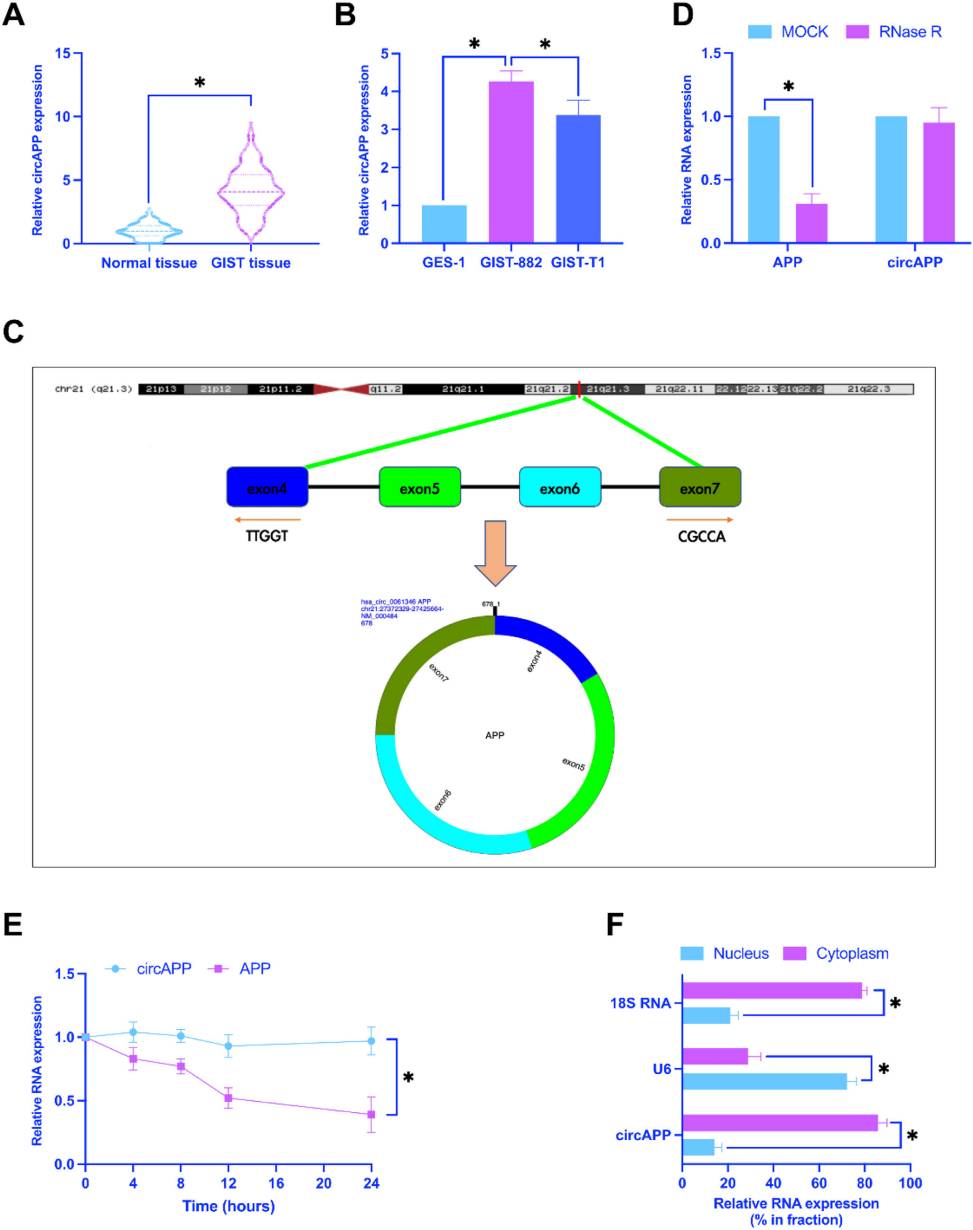
CircAPP is up-regulated in GIST. (A) CircAPP in GIST tissues and adjacent normal tissues, (B) CircAPP in GIST cell lines and normal cell lines, (C) Gene structure of circAPP on the circbase database, (D and E) RNaseR and actinomycin D treatment to explore the ring structure of circAPP, (F) Subcellular localization of circAPP; data are expressed as mean ± SD (*n* = 3). *p < 0.05.

CircAPP has been revealed to be abnormally expressed in GIST.^8^ Consistent results were also obtained in this study, and circAPP expression in GIST tissues and cell lines was higher than that in normal tissues and cells (Fig. 1A, B). The circbase database discovered that circAPP is 678 bp in length and consisted of exons 4‒7 of the APP gene located on chromosome 21q21.3 (Fig. 1C). Examinations of the ring structure of circAPP revealed that RNase R treatment only decreased linear APP mRNA but not circAPP (Fig. 1D). After actinomycin D treatment, circAPP had a longer half-life than linear APP mRNA (Fig. 1E). Subsequently, the location of circAPP in GIST-882 cells was examined by subcellular isolation assay, confirming circAPP mainly expressed in the cytoplasm (Fig. 1F).

### *Knockdown of circAPP inhibits GIST proliferation and the Warburg effect*

**Fig. 2:**
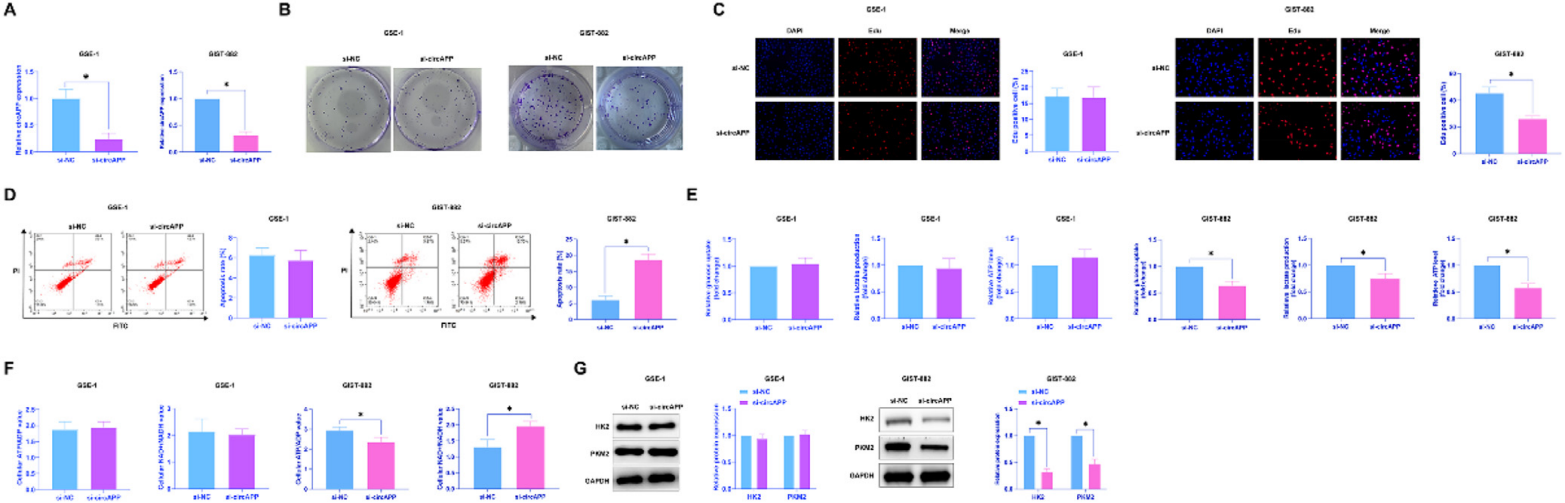
Knockdown of circAPP inhibits GIST proliferation and the Warburg effect. si-circAPP was transfected into GIST-882 and GES-1 cells. (A) Expression changes of circAPP, (B and C) Colony formation and EdU assays to assess cell proliferation, (D) Flow cytometry to analyze apoptosis, (E) Cellular glucose consumption, lactate production, and ATP levels, (F) ATP/ADP ratio and NAD^+^/NADH ratio, (G) Western blot to evaluate cellular HK2 and PKM2 protein expression; data are expressed as mean ± SD (*n* = 3). *p < 0.05.

Since GIST-882 has the highest expression level of circAPP, follow-up studies were performed using GIST-882. Focusing on the functions of circAPP in GIST, si-circAPP was transected into GIST-882 cells which maintained circAPP expression in a suppressed state (Fig. 2A). Corresponding to the decrease in circAPP expression, the cloning ability and Edu-positive cell number of GIST-882 cells examined by colony formation assay (Fig. 2B) and EdU assay (Fig. 2C) were reduced, and the apoptosis rate tested by flow cytometry was augmented (Fig. 2D). For examining Warburg effect in GIST-882 cells, glucose consumption, lactate production, and ATP levels, along with ATP/ADP and NAD^+^/NADH were the tested indices. In circAPP-silenced GIST-882 cells, reduction in glucose consumption, lactate production, ATP, and ATP/ADP, and upregulation of NAD^+^/NADH were detectable (Fig. 2E, F). Moreover, key Warburg effect proteins HK2 and PKM2 were examined by western blot: after silencing circAPP, HK2 and PKM2 levels were reduced (Fig. 2G), further proving the anti-Warburg effect property of silencing circAPP in GIST.

### *CircAPP has a binding relationship with miR-6838-5p*

**Fig. 3:**
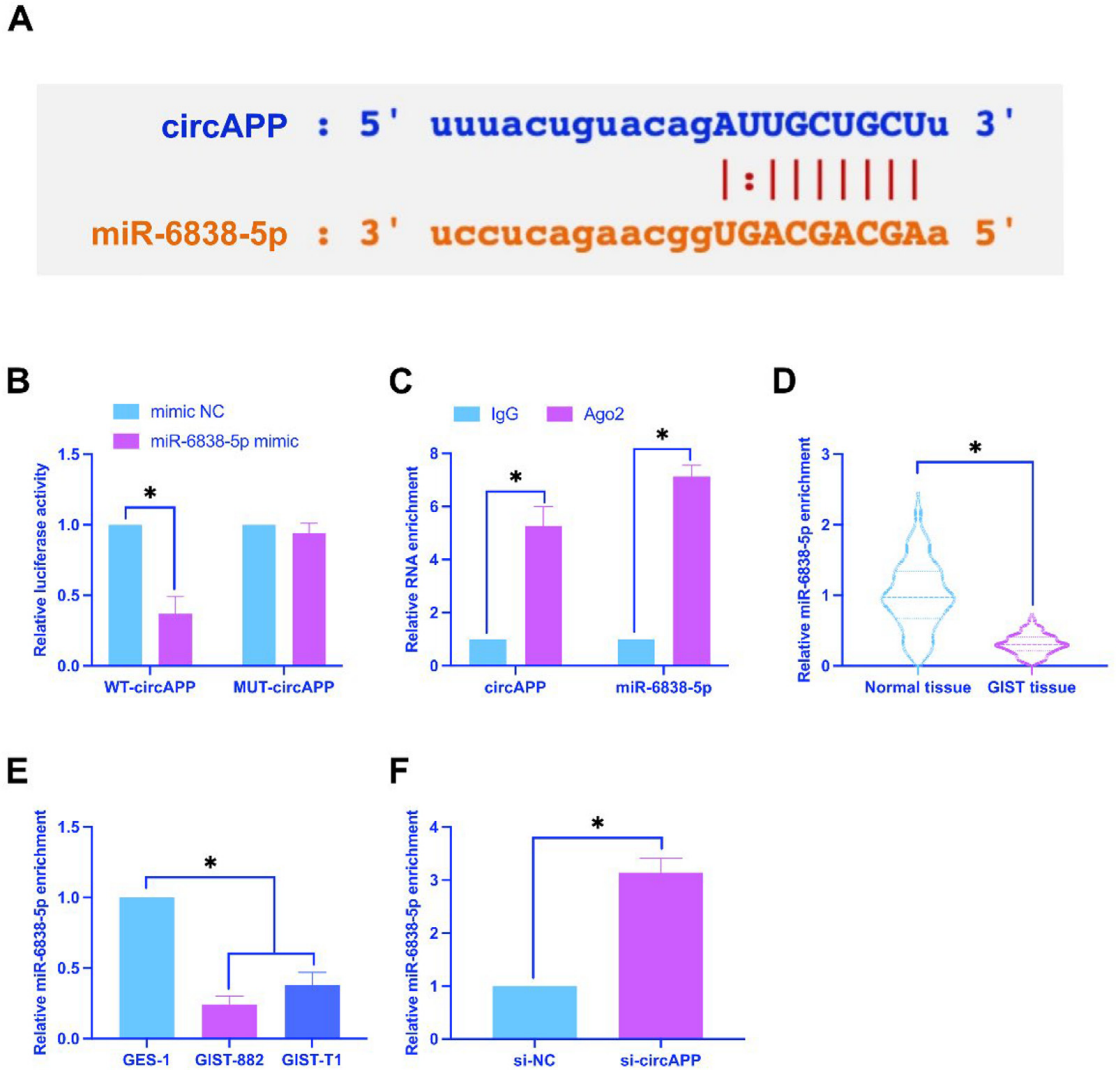
CircAPP has a binding relationship with miR-6838-5p. (A) Bioinformatics website to predict the potential targets of circAPP and miR-6838-5p, (B and C) Dual luciferase reporter assay and RIP assay to detect the targeting relationship between circAPP and miR-6838-5p, (D and E) miR-6838-5p expression in tissues and cells, (F) Effect of knockdown of circAPP on miR-6838-5p expression; data are expressed as mean ± SD (*n* = 3). *p < 0.05.

Through the prediction on the bioinformatics website https://starbase.sysu.edu.cn/, circAPP had a potential binding site for miR-6838-5p (Fig. 3A). Subsequently, this targeting relationship was confirmed, as the results claimed that luciferase activity decreased after co-transfection of WT-circAPP and miR-6838-5p mimic (Fig. 3B); miR-6838-5p and circAPP increased after Ago2 treatment (Fig. 3C). RNA gene analysis manifested the reduction of miR-6838-5p in GIST tissues and cells (Fig. 3D, E). Furthermore, the knockdown of circAPP in GIST-882 cells was also found to promote miR-6838-5p expression (Fig. 3F). Taken together, circAPP targets the regulation of miR-6838-5p levels.

### miR-6838-5p induces GIST proliferation and the Warburg effect

**Fig. 4:**
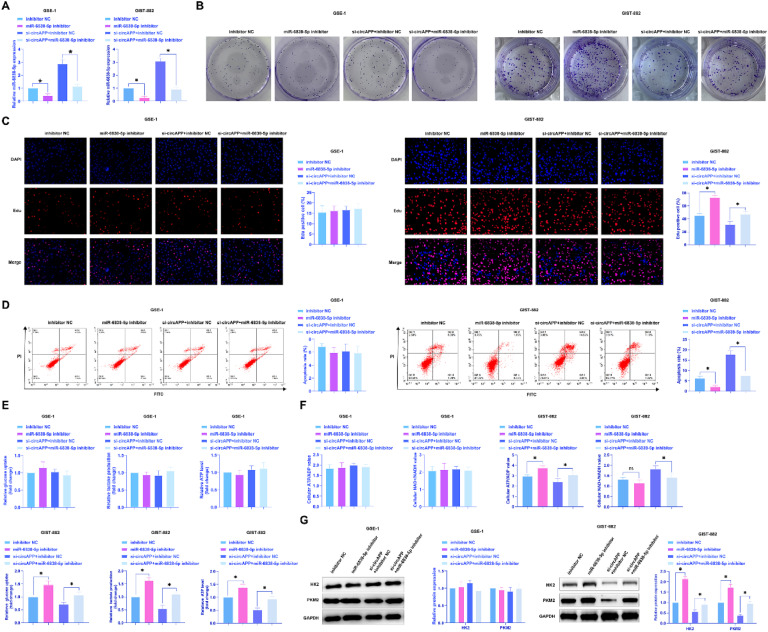
miR-6838-5p inhibitor promotes GIST proliferation and the Warburg effect and reverses the effect of si-circAPP. miR-6838-5p inhibitor was transfected alone or si-circAPP and miR-6838-5p inhibitor were co-transfected, (A) Expression changes of miR-6838-5p, (B and C) Colony formation and EdU assays to assess cell proliferation, (D) Flow cytometry to analyze apoptosis, (E) Cellular glucose consumption, lactate production, and ATP levels, (F) ATP/ADP ratio and NAD^+^/NADH ratio, (G) Western blot to evaluate cellular HK2 and PKM2 protein expression; data are expressed as mean ± SD (*n* = 3). *p < 0.05.

To confirm circAPP/miR-838-5p regulating the biological behavior of GIST, miR-6838-5p inhibitor and si-circAPP were transfected into GIST-882 cells. Transfection of si-circAPP alone miR-6838-5p expression, but the action of si-circAPP was impaired by miR-6838-5p inhibitor (Fig. 4A). Functional experiments showed (Fig. 4B‒G) that transfection of miR-6838-5p inhibitor alone enhanced the clonality and proliferation, decreased apoptosis rate, and promoted cellular glucose consumption, lactate production, ATP and ATP/ADP, downregulated NAD^+^/NADH, and induced HK2 and PKM2 protein expression. si-circAPP showed opposite effects which could be blocked by co-transfection of miR-6838-5p inhibitor. All in all, circAPP affects GIST proliferation and the Warburg effect by regulating miR-6838-5p expression.

### *CDV3 is modified by miR-6838-5p*

**Fig. 5:**
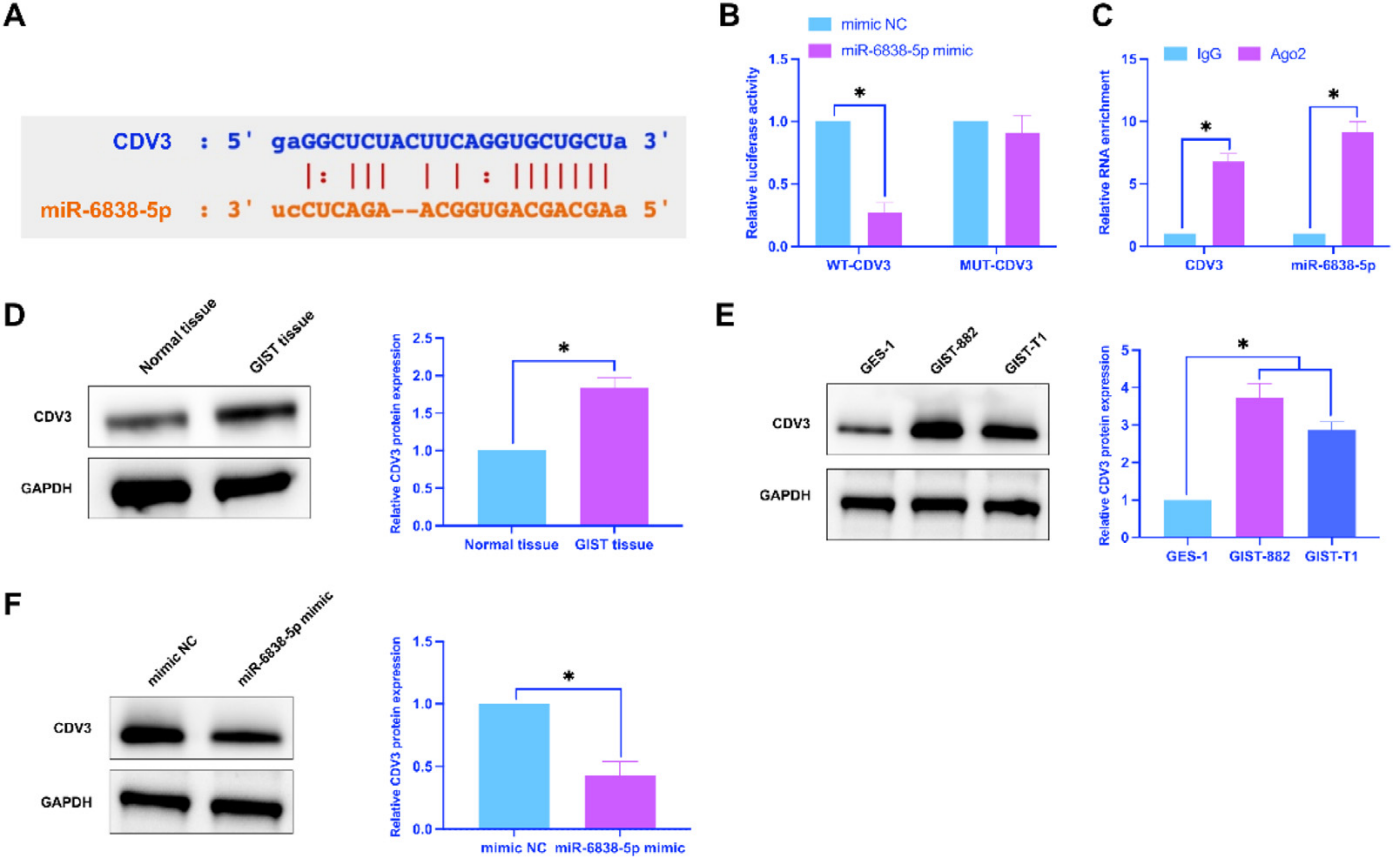
CDV3 is a target of miR-6838-5p. (A) Bioinformatics website to predict the potential targets of CDV3 and miR-6838-5p, (B and C) Dual luciferase reporter assay and RIP assay to detect the targeting relationship between CDV3 and miR-6838-5p, (D and E) CDV3 expression in tissues and cells, (F) Effect of miR-6838-5p on CDV3 expression; data are expressed as mean ± SD (*n* = 3). *p < 0.05.

The bioinformatics website starbase predicted the existence of potential binding sites for miR-6838-5p and CDV3 (Fig. 5A). Co-transfection of WT-CDV3 and miR-6838-5p mimic inhibited luciferase activity (Fig. 5B); CDV3 and miR-6838-5p were enriched with Ago2 (Fig. 5C). CDV3 was higher in both GIST tissues and cell lines than in normal tissues and cell lines (Fig. 5D, E). Furthermore, miR-6838-5p mimics suppressed CDV3 expression in GIST (Fig. 5F). Shortly, CDV3 is modified by miR-6838-5p.

### *CircAPP activates GIST proliferation and the Warburg effect by regulating the miR-6838-5p/CDV3 axis*

**Fig. 6:**
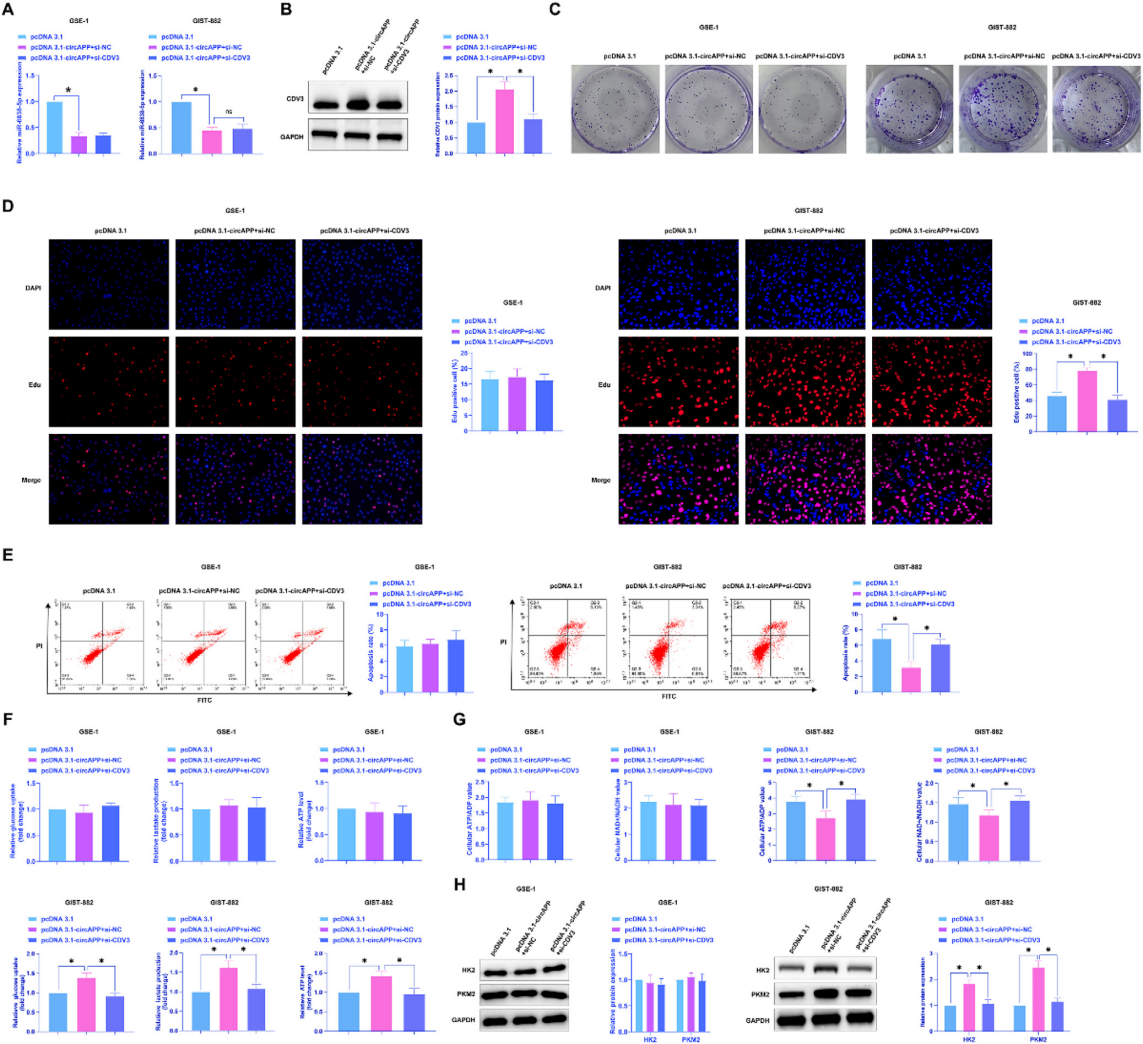
CircAPP promotes GIST proliferation and the Warburg effect by regulating the miR-6838-5p/CDV3 axis. pcDNA 3.1-circAPP and si-CDV3 were co-transfected, (A) Expression changes of miR-6838-5p, (B) Expression changes of CDV3, (C and D) Colony formation and EdU assays to assess cell proliferation, (E) Flow cytometry to analyze apoptosis, (F) Cellular glucose consumption, lactate production, and ATP levels, (G) ATP/ADP ratio and NAD^+^/NADH ratio, (H) Western blot to evaluate cellular HK2 and PKM2 protein expression; data are expressed as mean ± SD (*n* = 3). *p < 0.05.

To investigate circAPP/miR-6838-5p/CDV3 affecting GIST biological behavior, this study co-transfected pcDNA 3.1-circAPP and si-CDV3 into GIST-882 cells. Changes in miR-6838-5p and CDV3 expression were examined. pcDNA 3.1-circAPP inhibited miR-6838-5p and elevated CDV3 levels, while si-CDV3 had no effect on miR-6838-5p expression but reversed CDV3 expression (Fig. 6A, B). The functional rescue of pcDNA 3.1-circAPP by si-CDV3 was subsequently confirmed in the aspects of clonality, the number of Edu-positive cells, apoptosis rate, cellular glucose consumption, lactate production, ATP levels, ATP/ADP ratio, NAD^+^/NADH ratio, HK2 and PKM2 protein expression (Fig. 6C‒H). Taken together, circAPP promotes GIST proliferation and the Warburg effect via the miR-6838-5p/CDV3 axis.

### *CircAPP accelerates GIST tumor growth and metastasis*

**Fig. 7:**
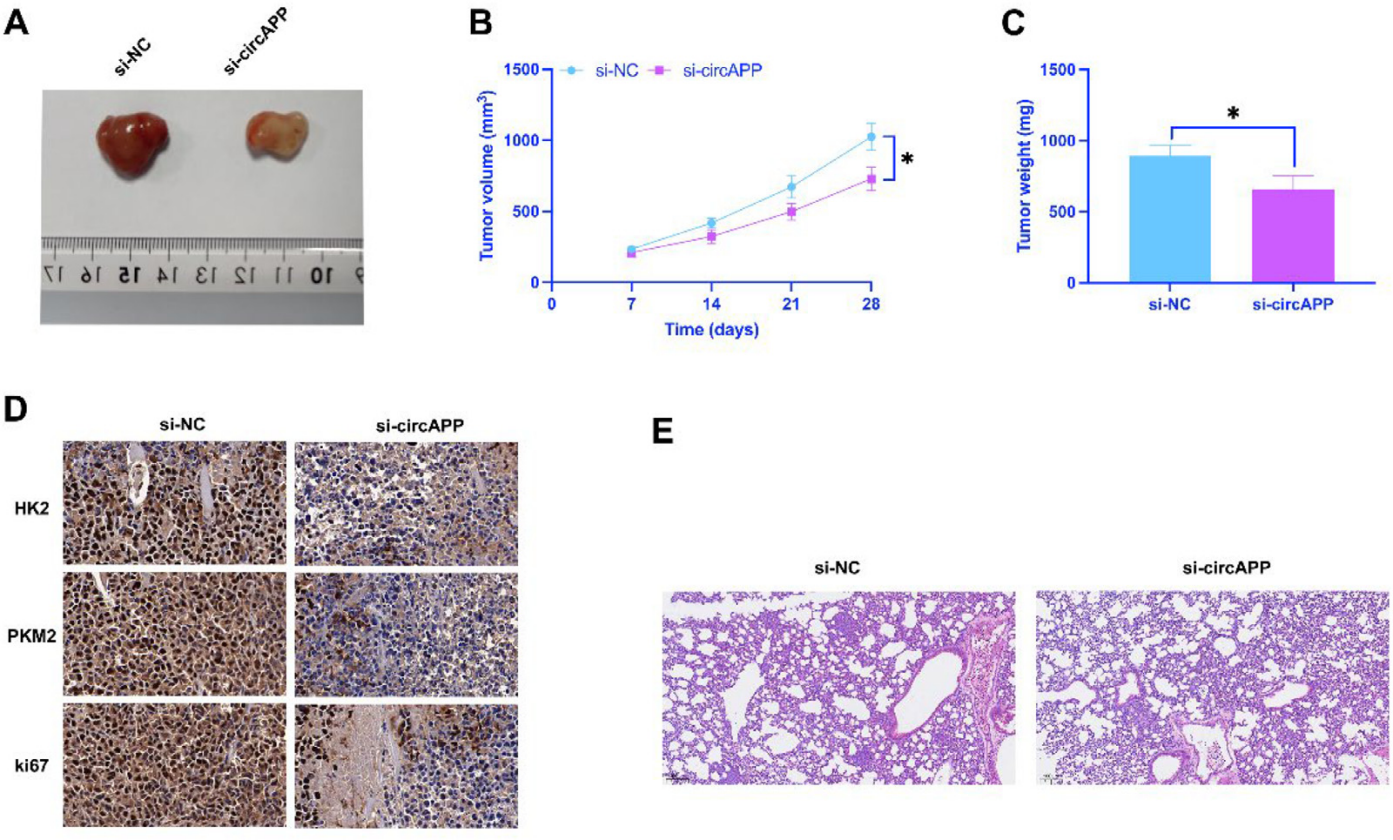
CircAPP inhibits GIST tumor growth and metastasis in vivo. (A) Representative image of tumors, (B) Tumor volume, (C) Tumor weight, (D) IHC image of HK2, PKM2, and Ki-67 proteins in tumors, (E) Western blot detection of tumor CDV3 expression, (F) HE images of tumor liver metastases; data are expressed as mean ± SD (*n* = 6). *p < 0.05.

This study examined the effect of circAPP on GIST tumor growth and liver metastasis *in vivo*. As demonstrated in Fig. 7A-C, ablating circAPP suppressed GIST tumor volume and weight. Immunohistochemistry showed that circAPP knockdown suppressed HK2, PKM2, and Ki-67 in GIST tumors (Fig. 7D). Furthermore, knockdown of circAPP suppressed CDV3 expression in tumors (Fig. 7E). Liver metastasis of GIST tumors was assessed by HE-staining (Fig. 7F), manifesting that circAPP depletion suppressed the number of metastatic nodules in the liver. circAPP mediates GIST tumor growth and metastasis.

## Discussion

GIST accounts for 1 %‒3 % of gastrointestinal tumors.^25^ CircRNAs are imperative regulators in various physiological processes^26^ and the occurrence of GIST.^27^ This study found that circAPP expression was augmented in GIST, and knocking down circAPP hampered GIST cell proliferation and the Warburg effect, triggered cell apoptosis, and limited GIST tumor growth and liver metastasis.

CircRNAs have become a research hotspot in recent years.^28^ As research progresses, the functions and mechanistic network of circRNAs in cancer have been implicated in tumor cell activities,^29^^,^^30^ including Warburg effect.^31^^,^^32^ The Warburg effect, proposed by Dr. Otto Heinrich Warburg in 1920, refers to the shift of tumor cell metabolism from oxidative phosphorylation to aerobic glycolysis induced by mitochondrial respiration damage.^33^ The Warburg effect is a central contributor to the mechanisms of cancer progression, contributing to the cellular progression of cancer cells and being involved in immune responses and drug resistance.^34^^,^^35^ Therefore, inhibiting the Warburg effect inhibits cancer progression. This study confirms that circAPP was a positive regulator of the Warburg effect. Similar to a previous report,^8^ this study also presented that circAPP was overexpressed in GIST. The *in vitro* results reported that downregulating circAPP inhibited GIST cell proliferation and the Warburg effect. Distant metastasis has been reported to be obstructive to cancer treatment.^36^ Therefore, this study further verified the suppressive impacts of silencing circAPP on GIST tumor growth and liver metastasis *in vivo*. miRNA sponging is the most well-studied mechanism of circRNA action, and circRNA competitively binds miRNA through complementary base pairing to inhibit the binding of miRNA to its target molecule, thereby regulating the expression of target Mrna.^37^ This study confirmed circAPP as a gene modifier of miR-6838-5p. miR-6838-5p is involved in a series of biological processes in cancer.^38^ miR-6838-5p is downregulated in osteosarcoma, and induction of miR-6838-5p suppresses tumor metastasis.^15^ In addition, miR-6838-5p is also involved in myocardial ischemia-reperfusion injury and cerebral hemorrhage injury.^39^^,^^40^ Here, this study implied that miR-6838-5p was lowly expressed in both GIST, and suppression of miR-838-5p mitigated the effect of silencing circAPP. This study further showed that, mechanistically, CDV3 was a target of miR-6838-5p. CDV3 was documented as an unidentified gene in breast cancer in 1999^41^ and it is overexpressed in hepatocellular carcinoma.^42^ This study also observed the abundance of CDV3 in GIST, and inhibition of CDV3 impaired circAPP restoration-mediated influences on GIST cell proliferation and the Warburg effect.

Although this study has partially elucidated the function of circAPP in GIST, further research is required in order to develop effective GIST treatments. On the one hand, due to the insufficient sample size, the correlation of circAPP with pathological T-staging and poor prognosis of GIST patients has not been established. On the other hand, the expression of most circRNAs is significantly correlated with GIST tumor size, mitotic number, and malignant degree by constructing circDNA expression profiles, and does not correlate with tumor site,^43^ but no experiments have been performed to verify this idea. The exact role of this still needs to be further explored, and the molecular expression of the circAPP/miR-6838-5p/CDV3 axis and whether its mechanism is affected by differences in primary sites is a direction for our future in-depth research. Despite its important role, miR-6838-5p is not the only target regulated by circAPP. Whether other miRNAs are involved in circAPP affecting Warburg effect of GIST and the related specific mechanism still needs to be further explored.

## Conclusion

This study demonstrates that a novel circRNA, circAPP, is upregulated in GIST tissues and cells and that upregulating circAPP expression promotes the Warburg effect by targeting the miR-6838-5p/CDV3 axis, which is of great significance for the malignant proliferation and metastasis of GIST. The results provide important data support for the future clinical treatment of GIST and the development of targeted drugs.

## Availability of data and materials

The datasets used and/or analyzed during the present study are available from the corresponding author upon reasonable request.

## Consent for publication

Written informed consent for publication was obtained from all participants.

## Ethical approval and consent to participate

All procedures performed in this study involving human participants were in accordance with the ethical standards of the institutional and/or national research committee and with the 1964 Helsinki Declaration and its later amendments or comparable ethical standards. All subjects were approved by the Affiliated Hospital of Inner Mongolia Minzu University (n° 201211Y62). Written informed consent was obtained from each subject.

The animal experiments were complied with the ARRIVE guidelines and performed in accordance with the National Institutes of Health Guide for the Care and Use of Laboratory Animals. The experiments were approved by the Institutional Animal Care and Use Committee of the Affiliated Hospital of Inner Mongolia Minzu University (n° 201306YT2).

## Authors’ contributions

GeTu ZhaoRi and ChunJuan Wang designed the research study. XianJing Zeng and JinHua Yuan performed the research. GeTu ZhaoRi and XianJing Zeng provided help and advice. ChunJuan Wang and JinHua Yuan analyzed the data. GeTu ZhaoRi and ChunJuan Wang wrote the manuscript. JinHua Yuan reviewed and edited the manuscript. All authors contributed to editorial changes in the manuscript. All authors read and approved the final manuscript.

## Funding

Not applicable.

## Declaration of competing interest

The authors declare no conflicts of interest.

## References

[1] Wang M, Devine C, Segaran N, Ganeshan D. (2021). Current update on molecular cytogenetics, diagnosis and management of gastrointestinal stromal tumors. World J Gastroenterol.

[2] Mu J, Sun X, Zhao Z, Sun H, Sun P. (2021). BRD9 inhibition promotes PUMA-dependent apoptosis and augments the effect of imatinib in gastrointestinal stromal tumors. Cell Death Dis.

[3] Mariño-Enríquez A, Ou W, Cowley G, Luo B, Jonker A, Mayeda M (2014). Genome-wide functional screening identifies CDC37 as a crucial HSP90-cofactor for KIT oncogenic expression in gastrointestinal stromal tumors. Oncogene.

[4] Johar D, Elmehrath A, Khalil R, Elberry M, Zaky S, Shalabi S (2021). Protein networks linking Warburg and reverse Warburg effects to cancer cell metabolism. Biofactors.

[5] Zhou W, Cai Z, Liu J, Wang D, Ju H, Xu R. (2020). Circular RNA: metabolism, functions and interactions with proteins. Mol Cancer.

[6] Zang J, Lu D, Xu A. (2020). The interaction of circRNAs and RNA binding proteins: an important part of circRNA maintenance and function. J Neurosci Res..

[7] Zhang Z, Yang T, Xiao J. (2018). Circular RNAs: promising biomarkers for human diseases. EBioMedicine.

[8] Zou F, Cao D, Tang Y, Shu L, Zuo Z, Zhang L. (2020). Identification of CircRNA-miRNA-mRNA regulatory network in gastrointestinal stromal tumor. Front Genet.

[9] Zhang M, Bai X, Zeng X, Liu J, Liu F, Zhang Z. (2021). circRNA-miRNA-mRNA in breast cancer. Clin Chim Acta.

[10] Zambello R, Cassatella M, Trentin L, Bulian P, Siviero F, Feruglio C (1991). Different mechanisms of activation of proliferating CD3+ cells in patients with lymphoproliferative disease of granular lymphocytes. Leukemia.

[11] Rupaimoole R, Slack F. (2017). MicroRNA therapeutics: towards a new era for the management of cancer and other diseases. Nat Rev Drug Discov.

[12] Iorio M, Croce C. (2012). MicroRNA dysregulation in cancer: diagnostics, monitoring and therapeutics. A comprehensive review. EMBO Mol Med.

[13] Chen Y, Qin C, Cui X, Geng W, Xian G, Wang Z. (2020). miR-4510 acts as a tumor suppressor in gastrointestinal stromal tumor by targeting APOC2. J Cell Physiol.

[14] Yun S, Kim W, Kwon Y, Jang M, Bauer S, Kim H. (2018). Survivin is a novel transcription regulator of KIT and is downregulated by miRNA-494 in gastrointestinal stromal tumors. Int J Cancer.

[15] Wang F, Sun H, Li K, Yang K, Xiang Y, Tian X. (2022). CircRASSF2 promotes IGF1R and osteosarcoma metastasis via sponging miR-6838-5p. Ann Transl Med.

[16] Zhou W, Ding X, Jin P, Li P. (2020). miR-6838-5p affects cell growth, migration, and invasion by targeting GPRIN3 via the Wnt/β-Catenin signaling pathway in gastric cancer. Pathobiology.

[17] Zhai X, Wu Y, Zhang D, Li H, Chong T, Zhao J. (2021). MiR-6838-5p facilitates the proliferation and invasion of renal cell carcinoma cells through inhibiting the DMTF1/ARF-p53 axis. J Bioenerg Biomembr.

[18] Zhou X, Yuan G, Wu Y, Yan S, Jiang Q, Sanyuan T. (2022). EIF4A3-induced circFIP1L1 represses miR-1253 and promotes radiosensitivity of nasopharyngeal carcinoma. Cell Mol Life Sci.

[19] Lu Y, Cheng J, Cai W, Zhuo H, Wu G, Cai J. (2021). Inhibition of circRNA circVPS33B reduces warburg effect and tumor growth through regulating the miR-873-5p/HNRNPK Axis in infiltrative gastric cancer. Onco Targets Ther.

[20] Du XF, Chen SL, Cui HT, Huang YM, Wang JR, Liu H (2022). Circular RNA hsa_circ_0083756 promotes intervertebral disc degeneration by sponging miR-558 and regulating TREM1 expression. Cell Prolif.

[21] Pu Z, Xu M, Yuan X, Xie H, Zhao J. (2020). Circular RNA circCUL3 accelerates the warburg effect progression of gastric cancer through regulating the STAT3/HK2 Axis. Mol Ther Nucleic Acids.

[22] Wu H, Zhao XB, Wang J, Jiang XY, Cheng Y, He YN (2022). Circular RNA CDR1as alleviates cisplatin-based chemoresistance by suppressing MiR-1299 in ovarian cancer. Front Genet.

[23] Seung B, Lim H, Shin J, Kim H, Cho S, Kim S (2018). CD204-Expressing tumor-associated macrophages are associated with malignant, high-grade, and hormone receptor-negative canine mammary gland tumors. Vet Pathol.

[24] He J, Chu Z, Lai W, Lan Q, Zeng Y, Lu D (2021). Circular RNA circHERC4 as a novel oncogenic driver to promote tumor metastasis via the miR-556-5p/CTBP2/E-cadherin axis in colorectal cancer. J Hematol Oncol.

[25] Qian X, Yan Y, Gao B, Wang W. (2020). Prevalence, diagnosis, and treatment of primary hepatic gastrointestinal stromal tumors. World J Gastroenterol.

[26] Song T, Xu A, Chen X, Gao J, Gao F, Kong X. (2021). Circular RNA circRNA_101996 promoted cervical cancer development by regulating miR-1236-3p/TRIM37 axis. Kaohsiung J Med Sci.

[27] Sheng H, Pan H, Yao M, Xu L, Lu J, Liu B (2021). Integrated analysis of circular RNA-Associated ceRNA network reveals potential circrna biomarkers in human breast cancer. Comput Math Methods Med.

[28] Yu T, Wang Y, Fan Y, Fang N, Wang T, Xu T (2019). CircRNAs in cancer metabolism: a review. J Hematol Oncol.

[29] Xie W, Liu L, He C, Zhao M, Ni R, Zhang Z (2021). Circ_0002711 knockdown suppresses cell growth and aerobic glycolysis by modulating miR-1244/ROCK1 axis in ovarian cancer. J Biosci.

[30] Wang X, Li R, Feng L, Wang J, Qi Q, Wei W (2022). Hsa_circ_0001666 promotes non-small cell lung cancer migration and invasion through miR-1184/miR-548I/AGO1 axis. Mol Ther Oncolytics.

[31] Chen Y, Song S, Zhang L, Zhang Y. (2021). Circular RNA hsa_circ_0091579 facilitates the Warburg effect and malignancy of hepatocellular carcinoma cells via the miR-624/H3F3B axis. Clin Transl Oncol.

[32] Liu A, Xu J. (2021). Circ_03955 promotes pancreatic cancer tumorigenesis and Warburg effect by targeting the miR-3662/HIF-1α axis. Clin Transl Oncol.

[33] Benny S, Mishra R, Manojkumar M, Aneesh T. (2020). From Warburg effect to reverse Warburg effect; the new horizons of anti-cancer therapy. Med Hypotheses.

[34] Vaupel P, Schmidberger H, Mayer A. (2019). The Warburg effect: essential part of metabolic reprogramming and central contributor to cancer progression. Int J Radiat Biol.

[35] Kitazawa M, Hatta T, Sasaki Y, Fukui K, Ogawa K, Fukuda E (2020). Promotion of the Warburg effect is associated with poor benefit from adjuvant chemotherapy in colorectal cancer. Cancer Sci.

[36] Koklesova L, Liskova A, Samec M, Zhai K, Abotaleb M, Ashrafizadeh M (2020). Carotenoids in cancer metastasis-status quo and outlook. Biomolecules.

[37] Gong Y, Jiao Y, Qi X, Fu J, Qian J, Zhu J (2022). Construction of a circRNA-miRNA-mRNA network based on differentially co-expressed circular RNA in gastric cancer tissue and plasma by bioinformatics analysis. World J Surg Oncol.

[38] Liu G, Wang P, Zhang H. (2019). MiR-6838-5p suppresses cell metastasis and the EMT process in triple-negative breast cancer by targeting WNT3A to inhibit the Wnt pathway. J Gene Med.

[39] Zhang G, Ding L, Sun G, Liu Z, Ou W, Wang B (2022). LncRNA AZIN1-AS1 ameliorates myocardial ischemia-reperfusion injury by targeting miR-6838-5p/WNT3A axis to activate Wnt-β/catenin signaling pathway. In Vitro Cell Dev Biol Anim.

[40] Jiang F, Liu X, Wang X, Hu J, Chang S, Cui X. (2022). LncRNA FGD5-AS1 accelerates intracerebral hemorrhage injury in mice by adsorbing miR-6838-5p to target VEGFA. Brain Res.

[41] Oh J, Grosshans D, Wong S, Slamon D. (1999). Identification of differentially expressed genes associated with HER-2/neu overexpression in human breast cancer cells. Nucleic Acids Res.

[42] Xiao H, Zhou B, Jiang N, Cai Y, Liu X, Shi Z (2018). The potential value of CDV3 in the prognosis evaluation in Hepatocellular carcinoma. Genes Dis.

[43] Zou FW, Cao D, Tang YF, Shu L, Zuo Z, Zhang LY. (2020). Identification of CircRNA-miRNA-mRNA regulatory network in Gastrointestinal Stromal Tumor. Front Genet.

